# Consequences of the impact of COVID-19 pandemic on breast cancer at a single Italian institution

**DOI:** 10.37349/etat.2022.00091

**Published:** 2022-07-29

**Authors:** Daniele Ugo Tari, Rosalinda Santonastaso, Fabio Pinto

**Affiliations:** 1 Department of Diagnostic Senology, District 12, Caserta Local Health Authority, 81100 Caserta, Italy; 2 Department of Economics, University of Campania “Luigi Vanvitelli”, 81043 Capua, Italy; 3 Department of Radiology, “A. Guerriero” Hospital, Caserta Local Health Authority, 81025 Marcianise, Italy; Università Politecnica Marche, Italy

**Keywords:** Breast cancer, coronavirus disease 2019, prevention, cancer care, diagnosis, screening, healthcare costs

## Abstract

**Aim:**

To evaluate the local impact of the coronavirus disease 2019 (COVID-19) pandemic on breast cancer (BC) care, with particular attention to the economical and psychological consequences of the possible delay of new diagnoses and treatments.

**Methods:**

Three years’ activity (from 2019 to 2021) has been compared. The number of BCs diagnosed from the total amount of mammographic and ultrasound (US) examinations performed in women aged more than 40 years old has been considered. A Pearson’s chi-squared test was performed to verify differences between results. Statistical significance was set at *P* ≤ 0.01.

**Results:**

A statistically significant difference was found in the number of BC diagnosed between screening and ambulatory care patients in both the 2019–2020 (χ^2^ = 24.93, *P* < 0.01) and 2019–2021 (χ^2^ = 29.93, *P* < 0.01) comparisons. No statistically significant difference was found in the data recorded between 2020 and 2021 (χ^2^ = 2.35, *P* > 0.01). By evaluating the specific age groups for each year, a statistically significant difference (*P* < 0.01) was found in the number of BC diagnosed in screening patients aged 50–69 years old in both 2019–2020 and 2019–2021 comparisons. The percentages of early BC diagnosed in 2019, 2020, and 2021 were 80.9%, 91.7%, and 89.8%, respectively. The adherence rates to screening in 2019, 2020, and 2021 were 67.5%, 45.2%, and 56.9%, respectively.

**Conclusions:**

Results showed a reduction of new diagnoses in the screening range during the pandemic in comparison with the previous period. The high percentage of early BC would seem to have prevented worsening outcomes. Nevertheless, women who have not undergone screening could present a more advanced stage disease in the following years. Consequently, the evaluation of possible solutions to guarantee an essential level of care with the purpose to avoid worsening patients’ outcomes and the increase in healthcare costs is mandatory.

## Introduction

Breast cancer (BC) is the most frequent cancer in women of all ages, with more than 55,000 new diagnoses every year in Italy [1]. Also, male BC should be considered because even if it is a rare entity, representing less than 1% of all male malignancies, its incidence has increased by 20–25% in the past few decades and continues to rise [2].

Italian national screening programs improved the prognosis of patients with BC, reaching a survival rate of 87% at 5 years (84% in Southern Italy), and gaining a significant reduction in the diagnosis of tumors in advanced stages (about 30%) [3]. This latter aspect determined a lower use of adjuvant or neo-adjuvant therapies, shorter surgery times with earlier return to work, and a consequential remarkable positive economic and psychological impact.

World Health Organization (WHO) declared the rapid universal spread of the coronavirus disease 2019 [COVID-19, severe acute respiratory syndrome coronavirus 2 (SARS-CoV-2)] infection as pandemic on March 11th, 2020 [4, 5]. On February 25th, 2022, WHO data reported 430,257,564 confirmed cases worldwide and 5,922,047 deaths from the beginning of the pandemic. On that date, 12,651,251 cases had been confirmed in Italy, including 154,013 deaths [5, 6]. After a period of stability, data showed a significant increase from middle December to early January due to the spread of the Omicron variant. Due to the COVID-19 emergency, hospital resources were redistributed from non-urgent to urgent care [7]. Consequently, Breast units suffered a significant reduction in their clinical activity with a potential delay in diagnostic evaluations and treatment for patients with BC. Moreover, the fear of contracting the infection has driven patients away from hospitals during the pandemic [8].

The literature investigating the association between delay and prognosis in patients with BC has yielded conflicting results [9], however, a short delay (e.g., 6–12 weeks) should not affect the overall outcome [10, 11]. Nevertheless, the periodic interruption of BC screening activities, following the spread of SARS-CoV-2 infection in the population, could have a considerable effect.

This paper aims to evaluate the local impact of the pandemic on BC care, with particular attention to the economical and psychological consequences of the possible delay of new diagnoses and treatments. Consequently, possible solutions are suggested to guarantee an essential level of care in similar extraordinary conditions.

## Materials and methods

At the Department of Diagnostic Senology, both screening and ambulatory examinations were performed. Asymptomatic women aged 50–69 years old were invited for a first-level examination through the Sani.A.R.P. web portal, which is a complex ecosystem of web-based software whose purpose is to collect and manage information about the health status of patients living in our territory [12]. Sani.A.R.P. database defined the target population of the screening program and a dedicated call center scheduled appointments to avoid overbooking. Overbooking is a practice routinely used in BC screening programs, but that has to be avoided in the pandemic era to allow the mandatory social distancing. Contrarily, women aged 40–49 years old and > 70 years old requiring a breast examination could directly book their appointment by contacting directly the call center of our department.

According to Caserta Local Health Authority diagnostic-therapeutic care pathway (DTCP) [13], a mammographic exam in double projection [medio-lateral-oblique (MLO) and cranio-caudal (CC)] using the digital breast tomosynthesis (DBT) technique with 2D synthetic reconstruction (s2D) has been performed for both screening and ambulatory care patients. According to Breast Imaging Reporting and Data System (BI-RADS) 5th edition [14], all women classified as category 3, 4, or 5 were sent to second-level examinations such as ultrasound (US) with cytological and/or histopathological examinations. All the women with lesions classified as category B3, B4, or B5 according to guidelines for non-operative diagnostic procedures and reporting in BC screening [15] at core needle biopsy (CNB) or vacuum-assisted breast biopsy (VABB) were sent to surgery or chemotherapy.

Three years’ activity (from 2019 to 2021) has been compared. The number of BCs diagnosed from the total amount of mammographic and US examinations performed in women aged more than 40 years old has been considered. All the lesions having undergone surgery with a confirmed post-surgical histopathological examination have been defined as BC. If the patient was considered inoperable (e.g., metastatic or locally advanced disease, comorbidity, etc.) or refused to undergo surgery, all the lesions classified as category B5 on the CNB have been considered as BC [15]. For statistical analysis, a Pearson’s chi-squared test was performed to verify differences between results. Statistical significance was set at *P* ≤ 0.01. Finally, the stage of BCs diagnosed from 2019 to 2021 and the adherence rate to screening between the three years have been compared.

## Results

In 2019, 6,056 DBTs have been performed. Of these, 5,725 (94.5%) come from screening asymptomatic patients aged 50–69 years old (group A) and 331 (5.5%) come from ambulatory care patients aged 40–49 and > 70 years old (group B). A total amount of 105 BCs have been found, 70 in group A and 35 in group B.

In 2020, 2,366 DBTs have been performed. Of these, 1,924 (81.3%) come from screening asymptomatic patients aged 50–69 years old (group C) and 442 (18.7%) come from ambulatory care patients aged 40–49 and > 70 years old (group D). A total amount of 60 BCs have been found, 39 in group C and 21 in group D.

In 2021, 3,104 DBTs have been performed. Of these, 2,364 (76.2%) come from screening asymptomatic patients aged 50–69 years old (group E) and 740 (23.8%) come from ambulatory care patients aged 40–49 and > 70 years old (group F). A total amount of 49 BCs have been found, 34 in group E and 15 in group F.

**Figure 1.**
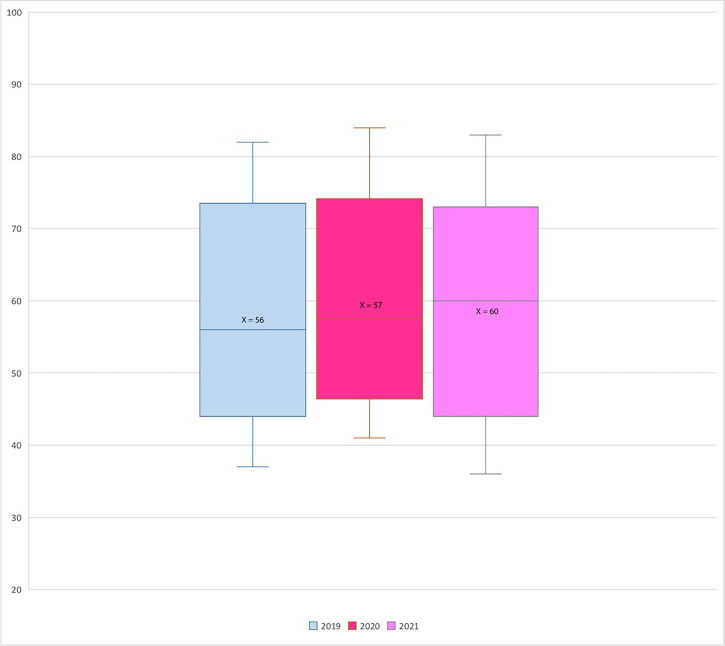
Box plot showing the distribution of BCs for ages per year. X-axis: years; Y-axis: number of BCs; X: mean value

Results and the overall population are reported in Table 1 and Table 2. The distribution and homogeneity of the population are showed in Figure 1.

**Table 1.** Total amounts of BCs diagnosed in 2019, 2020, and 2021 in the Department of Diagnostic Senology, District 12, Caserta Local Health Authority, divided for age ranges

**Years**	**2019**			**2020**			**2021**		
**Age**	**BC**	**Mean age (y)**	**SD**	**BC**	**Mean age (y)**	**SD**	**BC**	**Mean age (y)**	**SD**
40–49	21	45.9	± 3.4	11	46.4	± 2.6	10	47.8	± 8.0
50–69	70	57.9	± 5.9	39	58.2	± 5.3	34	59	± 5.1
> 70	14	75	± 4.1	10	76.1	± 4.8	5	76	± 4.3
Total	105	57.8	± 9.8	60	59.0	± 10.1	49	58.4	± 9.4

SD: standard deviation; y: year

**Table 2.** Number of examinations performed in the years 2019, 2020, and 2021 in the Department of Diagnostic Senology, District 12, Caserta Local Health Authority

**Years**	**2019**			**2020**			**2021**		
**Exams**	**Group A**	**Group B**	**Total**	**Group C**	**Group D**	**Total**	**Group E**	**Group F**	**Total**
DBT	5,725	331	6,056	1,924	442	2,366	2,364	740	3,104
US	241 (4.2%)	47 (14.2%)	288 (4.7%)	128 (6.6%)	37 (8.3%)	165 (6.9%)	137 (5.8%)	23 (3.1%)	160 (5.1%)
BC	70 (1.2%)	35 (10%)	105 (1.7%)	39 (2%)	21 (4.7%)	60 (2.5%)	34 (1.4%)	15 (2%)	49 (1.6%)

Group A, C, and E: women aged 50–69 years old. Group B, D, and F: women aged 40–49 and > 70 years old. The percentage reported in the US section represents the recall rate. The value in parenthesis in the BC section represents the percentage of BC diagnosed from the total DBT examinations performed

A statistically significant difference was found in the number of BC diagnosed in the two groups of patients (screening and ambulatory) in 2019–2020 (χ^2^ = 24.93, *P* < 0.01) and 2019–2021 (χ^2^ = 29.93, *P* < 0.01) comparisons. No statistically significant difference was found in the data recorded between 2020 and 2021 (χ^2^ = 2.35, *P* > 0.01). By evaluating the specific age groups for each year, a statistically significant difference (*P* < 0.01) was found in the number of BC diagnosed in screening patients aged 50–69 years old in 2019–2020 and 2019–2021 comparisons (Table 3).

**Table 3.** Statistical analysis of Pearson’s chi-squared test, divided for ages ranges

**Years’ comparison Age**	**2019–2020**		**2019–2021**		**2020–2021**	
**Age**	**χ^2^**	***P* value**	**χ^2^**	***P* value**	**χ^2^**	***P* value**
40–49	4.761905	< 0.05	5.761905	< 0.025	0.090909	< 0.9
50–69	13.72857	< 0.01	18.51429	< 0.01	2.016667	< 0.25
> 70	1.142857	< 0.75	5.785714	< 0.025	2.5	< 0.25
Total	19.28571	< 0.01	29.86667	< 0.01	2.016667	< 0.25

Statistical significance was set at *P* ≤ 0.01

The stage and percentage of BCs diagnosed from 2019 to 2021 are reported in Tables 4 and 5. In particular, the percentages of early BC diagnosed in 2019, 2020, and 2021 were 80.9%, 91.7%, and 89.8%, respectively. The adherence rates to screening in 2019, 2020, and 2021 were 67.5%, 45.2%, and 56.9%, respectively (Table 6).

**Table 4.** BC staging in the years 2019, 2020, and 2021

**BC stage**		**Early BC**					**Locally advanced BC**					**Metastatic BC**		**BC**
**Years**	**Age**	**0**	**IA**	**IIB**	**IIA**	**Total**	**IIB**	**IIIA**	**IIIB**	**IIIC**	**Total**	**IV**	**Total**	**Total**
2019	40–49	2	12	0	5	19 (90.5%)	0	0	1	1	2 (9.5%)	0	0	21
	50–69	15	30	2	11	58 (82.9%)	4	2	2	2	10 (14.3%)	2	2 (2.9%)	70
	> 70	3	3	1	1	8 (57.1%)	1	0	0	0	1 (7.1%)	5	5 (35.7%)	14
2020	40–49	4	5	1	0	10 (90.9%)	0	0	1	0	1 (9.1%)	0	0	21
	50–69	11 (2*)	18 (2*)	1	6	36 (92.3%)	1	0	1	0	2 (5.1%)	1	1 (2.6%)	39
	> 70	3	2	1	3	9 (90%)	1	0	0	0	1 (10%)	0	0	10
2021	40–49	3	4 (1*)	0	1	8 (80%)	1	0	0	0	1 (10%)	1	1 (10%)	10
	50–69	10 (1*)	19 (5*)	0	2 (1*)	31 (91.2%)	1	0	1	0	2 (5.9%)	1	1 (2.9%)	34
	> 70	1	4 (1*)	0	0	5 (100%)	0	0	0	0	0	0	0	5

The value in parenthesis represents the percentage of early BC, locally advanced BC, and metastatic disease from the total of BCs diagnosed.

*: number of patients who refused to undergo surgery; for these patients, we considered the result of CNB and the extension of disease at breast imaging [15]

**Table 5.** Number and percentage of BCs diagnosed from 2019 to 2021, divided per stage

**Years**	**Early BC**	**Locally advanced BC**	**Metastatic BC**	**Total**
2019	85 (80.9%)	13 (12.4%)	7 (6.7%)	105
2020	55 (91.7%)	4 (6.7%)	1 (1.7%)	60
2021	44 (89.8%)	3 (6.1%)	2 (4.1%)	49

**Table 6.** Adherence rate to screening from 2019 to 2021

**Years**	**Women invited**	**Women screened**	**Adherence rate**
2019	8,481	5,725	67.5%
2020	4,251	1,924	45.2%
2021	4,150	2,364	56.9%

## Discussion

BC is a disease with a significant social and economic impact on the entire community. It has long-lasting effects and the National Health Service is not always able to cover all its costs. Therefore, patients and family members often have to face them also with their own resources.

Focusing on people with cancer, after five years from the diagnosis, the social cost is € 36.4 billion per year in total. Of these, over € 5.8 billion are related to direct expenses and over € 30 billion to indirect costs (for example income losses, cessation of work activity, etc.). The monetary value of assistance and/or surveillance activities directly provided is € 12 billion [16].

Recent studies evaluating breast unit activity in 2020 compared to 2019, reported an increased number of referrals for both diagnostic exams in suspected BC patients (28%) and patients who received their first treatment for a BC diagnosis (16%) [17]. Jacob et al. [18] showed a significant reduction in new cancer diagnoses in Germany between March and May of 2020 compared with 2019. De Vincentiis et al. [19] reported that the incidence of BC decreased by 26% in 2020 in Italy, while Kaufman et al. [20] showed a decrease of 51.8% in the USA.

Results of this paper showed a reduction of new diagnoses in the screening range during the pandemic in comparison with the previous period. This reflected a massive decrease in the number of mammograms performed among 50–69 years old asymptomatic women. Even if the small number of patients could represent a major limit, the homogeneity of the population made our results quite realistic (Figure 1). Therefore, a possible delayed diagnosis, especially in the screening age range, could have determined the identification of a BC at a latter stage, with consequent more invasive breast surgery or neoadjuvant or adjuvant therapy, and a worse patient outcome with an increase in healthcare costs in total.

Data reported in Tables 4 and 5 expressed a remarkable efficacy of our activity due to the high rate of early BCs diagnosed (especially stage 0 and stage IA). Nevertheless, it is important to underline the overall reduction of examinations in the screening and over 70 population, wherein the percentage of locally advanced BC or metastatic disease (secondary cancer) is greater. Furthermore, the diagnosis of secondary cancer in a woman aged < 50 years old in 2021 and the progressive increase in the number of patients who refused to undergo surgery from 2020 to 2021 (respectively 4 and 9) is also worthy of attention (Table 4).

Primary and secondary cancer have different economic impacts. Indeed, the economic burden of the patient with secondary cancer (0.8%) is higher than that of patients with primary cancer (99.2%), accounting for an increase of 7% of direct costs and of 11% of average social security costs per patient/year. This aspect is not negligible in terms of prevention. An early diagnosis not only allows a better prognosis but also reduces the economic burden because of early access to care for the patient himself. Better treatment of primary tumors could also lead to a significant reduction in spending. On the contrary, chemotherapy significantly affects both direct and indirect costs. Researchers estimated a productivity loss related to chemotherapy of € 158 million, of which about € 33 million for short-term absences and € 118 million for prolonged absences [21]. The “financial toxicity” for individual patients has a total weight of € 4.7 million and € 1,236 per year per person, on average. Furthermore, the loss of emotional well-being accounts for about € 92 million. The most affected by “financial toxicity” are patients living in Southern Italy, non-working, and aged between 35–44 years. Women lose days of work or study twice as often as men do. Finally, patients have to face a total amount of almost € 5 billion/year not covered by the National Health System. Of these, the highest costs are those for medical visits (€ 1.1 billion), transport and stays at the place of care (€ 1.5 billion), drugs (€ 808 million), and reconstructive surgery (€ 425 million) [21].

The Italian National Screening Observatory (ONS) [22] reported that in the first 9 months of 2020, compared to 2019, there was a reduction of 947,322 (–34.5%) invitations with great variability between regions. Furthermore, a strong difference emerged by distinguishing the first 5 months (–41.6%) from the subsequent period (19.6%). Indeed, from January to September 2020, over 600,000 fewer women underwent screening mammography compared to the same period in 2019 (–43.5%). In particular, from June to September, there has been a marked slowdown in the proportional decrease in the exams performed, going from –53.6% (January–May) to –28.1% (June–September). This aspect was probably related to an attempt to recovery from the delay. The relationship between the percentage of decrease of the women invited and the exams performed between 2020 and 2019 has given us an indication of the propensity to participate in the BC screening program. This ratio for mammography screening was 0.79 (34.5%/43.5%). This means that, compared to 2019, in 2020 the propensity to participate has decreased relatively by 21%. The number of undiagnosed cancers is estimated to be 2,793. At the end of May, the estimate was 2,099 cases [22]. In our department, the adherence rates in 2019, 2020, and 2021 were 67.5%, 45.2%, and 56.9%, respectively (Table 6). According to the aforementioned evaluations, the difference between the first two years could reflect the reduction in the propensity to participate in screening caused by the pandemic combined with the reduction of the activity during lockdown, while the increase in the adherence rate of 2021 could reflect the attempt to recover the delay in the latter period. The reduction in the number of new BC diagnoses during the pandemic could be caused also by the patients’ fear of contracting SARS-CoV-2 infection, probably due to the belief that hospitals are infectious reservoirs [18, 23]. Indeed, 13 women refused to undergo surgery in 2020 and 2021 (4 and 9, respectively) in our department (Table 4).

According to this scenario, triaging both patients’ clinical status and urgent clinical cases, and awake and fast surgery have been mandatory to optimize the allocation of the limited resources during higher peaks, increasing the number of treated patients and reducing hospitalizations as well as the risk of cross-infection [24].

COVID-19 pandemic has created long-term and permanent challenges that will change the practice of medicine in the future. Currently, one of the major challenges continues to be the maintenance of social distancing, which may not be possible in various settings, such as in waiting rooms and when performing common procedures.

Given these considerations, the general population should be better informed that the risk of contamination is relatively low when all effective protective measures are respected [25].

Furthermore, the implementation of better DTCP could be a significant resource also to cut costs. Indeed, in an ideal system, the remuneration should occur for pathology rather than for single access to the service. Some experiences have been carried out in diabetology [26], but this remains a path to be explored in all chronic pathologies and, in particular, in oncology where the commitment of resources is very relevant and where timeliness and clinical, therapeutic and organizational appropriateness becomes crucial.

Therefore, we proposed a model to manage patients and healthcare staff in order to give continuity in the provision of an essential level of care in BC screening and ambulatory settings, contracting the waiting lists, and reducing the rate of advanced BC [27]. This protocol separated patients into three different clinical scenarios by performing a telephone questionnaire before each diagnostic exam or a nasopharyngeal swab before every recovery or invasive diagnostic procedure and demonstrated usefulness in optimizing resources.

Finally, during the COVID-19 pandemic, there has been a large use of telemedicine. Indeed, virtual consultations would allow non-urgent patients to be seen and have any concerns addressed in a more efficient manner, freeing up resources for urgent patients. Nevertheless, although telemedicine provides convenience in some areas, it does not allow for to preservation of relevant aspects of in-hospital activity, such as physical examination, the establishment of patient-clinician rapport on the basis of body language as well as staff relationships and teamwork [28].

## Abbreviations

BC: breast cancer
CNB: core needle biopsy
COVID-19: coronavirus disease 2019
DBT: digital breast tomosynthesis
SARS-CoV-2: severe acute respiratory syndrome coronavirus 2
US: ultrasound

## Declarations

### Availability of data and materials

The datasets analyzed for this study can be found in saniarp.it, the Caserta Local Health Authority reporting database, and from the register of our daily activities.

### Copyright

© The Author(s) 2022.
